# Does Malpractice Risk Deter Medical Students From Pursuing Obstetrics and Gynecology?

**DOI:** 10.7759/cureus.82710

**Published:** 2025-04-21

**Authors:** An Thai, Kimberly Huynh, Trevor Pickering, Angela Maron, Lana Wang, Hindi Stohl

**Affiliations:** 1 Obstetrics and Gynecology, Western University of Health Sciences, Pomona, USA; 2 Obstetrics and Gynecology, Harbor University of California Los Angeles Medical Center, Torrance, USA; 3 Public Health Sciences, University of Southern California, Los Angeles, USA; 4 Obstetrics and Gynecology, Harbor UCLA (University of California, Los Angeles) Medical Center, Los Angeles, USA

**Keywords:** choosing speciality, fear of malpractice, medical residency, medical school students, medico-legal implications, obstetrics and gynecology residency, obstetrics & gynecology, osteopathic medical school

## Abstract

Background

This research addresses the intricate process through which medical students choose or reject a specialty by investigating the factors that influence their decisions. One such factor is malpractice risk, which is a substantial consideration in specialties with higher risk for malpractice litigation, such as obstetrics and gynecology (Ob/Gyn). Our research probes the relationship between medical students’ perception of risk for malpractice litigation and specialty preference by exploring the factors that deter medical students from choosing the field of Ob/Gyn and elucidating the factors that impact specialty choice.

Methods

Conducted as an online survey via email, our research engaged medical students across all four graduating class years (Class of 2022 to 2025) at Western University of Health Sciences Pomona and Western University of Health Sciences of Northwest osteopathic medical schools. There is a ratio of 75 males to every 100 females per graduating class (Class of 2022 to 2025). Rigorous beta testing and multiple revisions preceded the survey. Reminder emails were sent at 1- and 3-week intervals after distribution. We employed Pearson’s Chi-square tests and Fisher’s exact test to identify factors influencing specialty choice and assess perceived medico-legal risk.

Results

We obtained responses from 392 medical students across all four classes. The total respondents were 150 males and 242 females. Our analysis revealed that risk for malpractice litigation ranked as the third most prevalent factor dissuading medical students from pursuing Ob/Gyn; however, gender-specific disparities among other factors also played a role. Males cited disinterest, female predominance, and patient population as reasons against choosing Ob/Gyn, while females emphasized lifestyle and competitiveness. The study underscored factors pivotal for those choosing Ob/Gyn, including patient-related aspects and procedural abilities.

Conclusions

Medical liability did not significantly influence osteopathic medical students already planning to pursue Ob/Gyn, whereas gender emerged as a key factor among those deterred. Male students demonstrated greater awareness of medico-legal practice laws, though both genders reported only limited knowledge. Among those not choosing Ob/Gyn, females cited competitiveness, liability concerns, and lifestyle as significant deterrents. These gender-specific differences highlight disparities in medical education and underscore the need for targeted mentorship and educational initiatives to foster diversity within the specialty. Understanding these factors can help medical schools support students' specialty decisions and develop strategies to cultivate a more balanced workforce.

## Introduction

Medical students are tasked with the difficult decision of choosing which specialty they hope to pursue before their medical school journey ends. A study by Katz et al. [1] revealed that 84% of medical students change their career selection due to a negative factor in their initial choice. Recognizing the myriad factors that influence each medical student’s decision is crucial for medical education and residency recruitment. This insight is invaluable for sharing with current medical students who might not be aware of the diverse facets of their chosen specialty before applying to residency. 

Regarding obstetrics and gynecology (Ob/Gyn) residency, selection factors that were considered important in fourth-year medical students who applied for Ob/Gyn included overall surgical training, laparoscopic training, exposure to maternal fetal medicine, and exposure to gynecological surgery [2,3]. Some negative factors observed in Ob/Gyn residents include long work hours, unsafe work environments, and medical litigations. In fact, in a study by Katz et al. [1], students who chose high-risk specialties like Ob/Gyn were more likely to have considered the probability of a malpractice suit and halting medically needed high-risk procedures due to fear of being sued. However, it was interesting to find that even when aware of the malpractice climate, this did not discourage students from going into high-risk specialties. While many medical students entering Ob/Gyn and other high-risk specialties understand the repercussions of medical malpractice, the depth of knowledge and the effects it can have on one’s personal well-being are not fully realized until it happens [3,4]. It has been found that malpractice climate and adverse litigation influence were significant factors for medical students switching from a high-risk specialty of choice, such as Ob/Gyn, to a specialty at lower risk of being sued [5]. However, an additional study found that students who chose Ob/Gyn as a medical specialty were not affected by malpractice concerns. Some studies suggest that it serves as a significant deterrent, prompting medical students to consider alternative paths [6-10]. Conversely, other research findings argue against its substantial influence. Additionally, there are studies that explore potential gender-based differences in perception, though not all investigations support this dichotomy. Overall, the existing literature on medicolegal risk for students pursuing Ob/Gyn presents a nuanced picture, indicating that its impact varies.

Do medical students’ perceptions of malpractice affect their choice of specialty? For those who do not choose to pursue Ob/Gyn, what are the main factors that detract them from the field? What factors are ultimately important when deciding on a specialty? We surveyed Southern California medical students at various points in their medical education about their perceptions of malpractice, various factors that contribute to their choice in specialty, and their interest in obstetrics and gynecology.

## Materials and methods

Methods

For this institutional review board-approved study, we developed a survey after reviewing the literature published on this subject. We then performed beta testing and underwent three rounds of beta testing for clarity and reliability. Following these steps, we conducted an online survey of medical students (n = 1,200) across all four graduating class years of one Southern California medical school. The survey targeted 1,200 medical students from the graduating classes of 2022 to 2025. Participation was voluntary and anonymous, with no identifying information collected. Reminder e-mails were sent at one- and three-week intervals, encouraging students to complete the anonymous survey, with four weeks after initial distribution set as the deadline to complete the survey.

Survey responses were analyzed using IBM SPSS Statistics for Windows, Version 28 (Released 2021; IBM Corp., Armonk, New York). Pearson’s chi-square test was utilized for survey questions with four or more response options, while Fisher’s exact test was used for those with three or fewer response options.

The survey examined various demographics, including gender (male, female, other), graduating class year (2022-2025), and preferred practice setting (rural, suburban, urban). It also explored participants' career preferences and the reasons behind their choices at the time of the survey, as well as their sources of knowledge about medical liability. Questions were also asked regarding understanding of medical malpractice laws (anchors: none, slight, some, moderate, comprehensive); factors that influenced the selection of a medical specialty, which were based on a three-point response scale (anchors: minor factor, non-factor, major factor); and reasons for not pursuing Ob/Gyn, including length of training, hours worked in residency, competitiveness of matching, patient population, disinterest in the field, liability risk, disinterest in surgery, work-life balance, female predominance in the field, and a negative Ob/Gyn clerkship experience. Survey responses were analyzed using either Pearson’s chi-square test or Fisher’s exact test. For survey questions with four or more response options, Pearson’s chi-square test was utilized. For questions with three or fewer response options, Fisher’s exact test was utilized.

## Results

The survey was distributed to students from the graduating classes of 2022, 2023, 2024, and 2025 at Western University, of which 392 responses were collected. Of these, 61.7% (n = 242) were female and 38.3% (n = 150) were male. The top three factors deterring students from choosing Ob/Gyn were lifestyle (61.5%), disinterest in the field (61.2%), and medical liability concerns (36.9%) (Table 1).

**Table 1:** Factors deterring medical students from pursuing Ob/Gyn, overall and by gender *Pearson’s chi-squared test.

Characteristic	Overall (N=392), n (%)	Female (N=242), n (%)	Male (N=150), n (%)	p-value*
Lifestyle	192 (61.5%)	125 (69.4%)	67 (50.8%)	<0.001
Disinterest in the field	191 (61.2%)	83 (46.1%)	108 (81.8%)	<0.001
Medical liability	115 (36.9%)	78 (43.3%)	37 (28.0%)	0.006
Disinterest in procedures	110 (35.3%)	64 (35.6%)	46 (34.8%)	0.9
Competitiveness	85 (27.2%)	72 (40.0%)	13 (9.85%)	<0.001
Patient population	75 (24.0%)	31 (17.2%)	44 (33.3%)	0.001
Female predominance	55 (17.6%)	8 (4.44%)	47 (35.6%)	<0.001
Length of residency	33 (10.6%)	23 (12.8%)	10 (7.58%)	0.14

At this time, most respondents reported that they planned to pursue internal medicine, family medicine, pediatrics, and emergency medicine. Among the respondents, the fifth and sixth most popular specialties were surgery/surgical subspecialties and Ob/Gyn, respectively.

**Figure 1:**
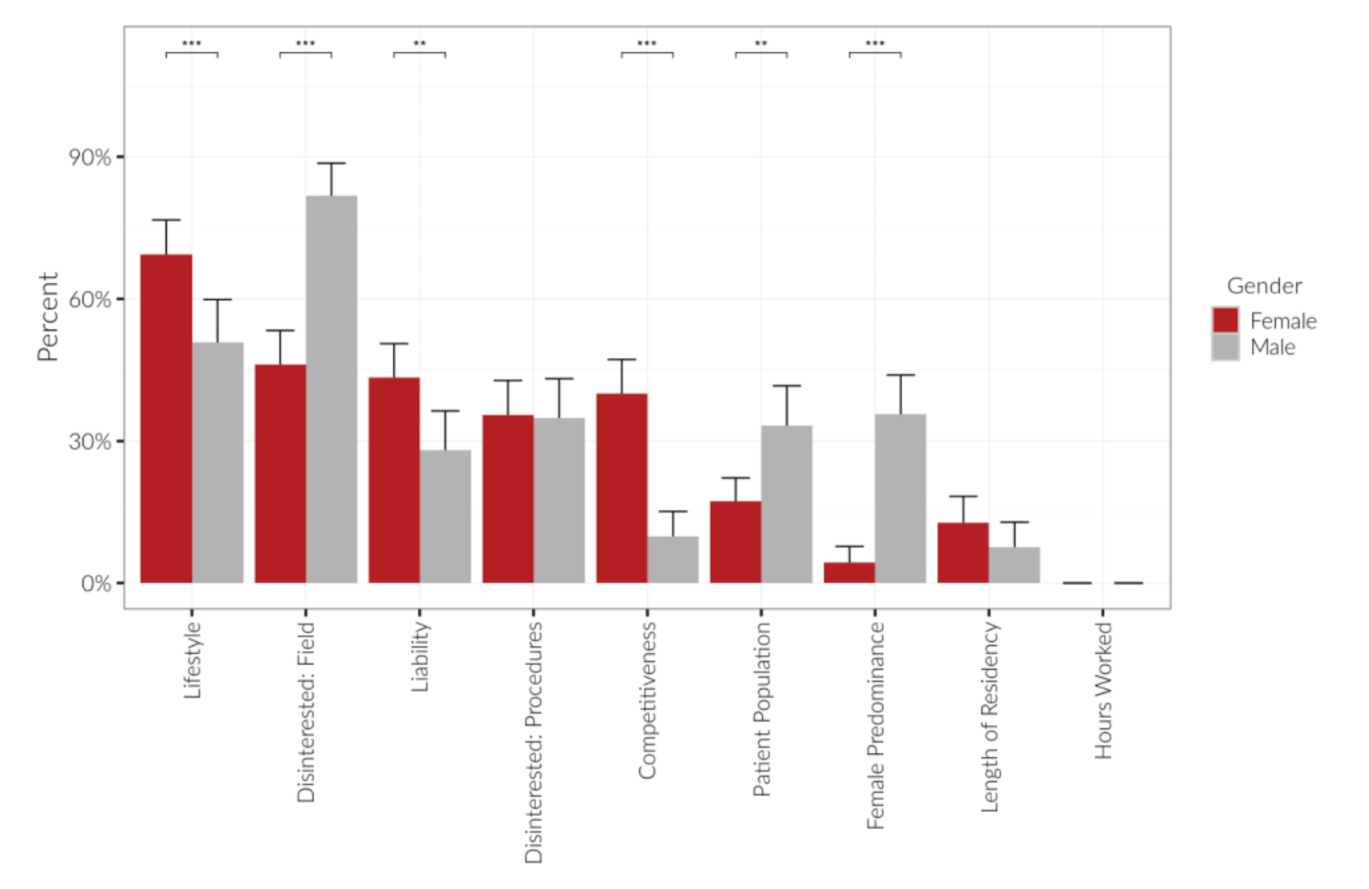
Comparison of medical students’ deterring factors from Ob/Gyn by gender The most common responses as to why medical students were disinterested in Ob/Gyn were “lifestyle”, “disinterest in the field”, “liability”, and “disinterest in procedures”, which remained consistent across genders. The factors with asterisks (*) show a statistically significant difference between male vs. female medical students. Pearson's chi-squared test was used.

Medical liability concerns differed by gender, with a statistically significantly higher proportion of female students (43.3%) reporting liability as a deterrent compared to males (28.0%) (p = 0.006). Additionally, competitiveness was cited as a barrier more frequently by female students (40.0%) than males (9.85%) (p < 0.001), while male students more often cited patient population (33.3%) and female predominance (35.6%) as deterrents (p = 0.001 and p < 0.001, respectively) (Figure 1).

When asked about their knowledge of medical liability, 41% of the students surveyed reported having had no education regarding medical malpractice, while the second highest percentage (27.1%) reported learning about medical liability through their professors, as shown in Table 2. Interestingly, concerns about malpractice risk and litigation did not heavily influence those already committed to pursuing Ob/Gyn. However, gender played a notable role in deterring others from the field. Male students showed slightly higher awareness of medico-legal practice laws, although their understanding was generally limited, classified as “slight” or “some” knowledge across genders. For female students opting out of Ob/Gyn, key deterrents included the specialty’s competitiveness, perceived liability risks, and lifestyle considerations. To better gauge the respondents’ depth of knowledge about malpractice litigation, their levels of understanding were further examined, with findings presented in Table 3.

**Table 2:** Medical students' experience of how they learned about medical liability Summary of students’ responses to “How did you learn about medical liability?” separated by gender. Students’ responses to multiple choice questions included “clinical experiences”, “have not learned”, “past education”, “personal experiences”, “peers”, and “professors”. Data are given as the number of respondents and percentages of respondents in parentheses, with each column totaling 100%. Pearson's chi-squared test was used.

How did you learn about medical liability?	Overall	Female	Male	p-value
Clinical experiences	79 (22.5%)	46 (21.3%)	33 (24.4%)	0.5
Have not learned	146 (41.6%)	94 (43.5%)	52 (38.5%)	0.4
Past education	39 (11.1%)	20 (9.26%)	19 (14.1%)	0.2
Past job	83 (23.6%)	49 (22.7%)	34 (25.2%)	0.6
Personal experiences	31 (8.83%)	15 (6.94%)	16 (11.9%)	0.11
Peers	58 (16.5%)	24 (11.1%)	34 (25.2%)	<0.01
Professors	95 (27.1%)	62 (28.7%)	33 (24.4%)	0.4

**Table 3:** Medical students' own understanding of malpractice litigation Summary of students’ responses to “What is your understanding of malpractice litigation?” separated by gender. Students rated their malpractice understanding as “none”, “slight”, “some”, “moderate”, or “comprehensive”. Pearson's chi-squared test was used.

Malpractice understanding	Overall	Female	Male
None	126 (36.0%)	95 (44.2%)	31 (23.0%)
Slight	153 (43.7%)	81 (37.7%)	72 (53.3%)
Some	63 (18.0%)	36 (16.7%)	27 (20.0%)
Moderate	6 (1.71%)	3 (1.40%)	3 (2.22%)
Comprehensive	2 (0.57%)	0 (0%)	2 (1.48%)

**Figure 2:**
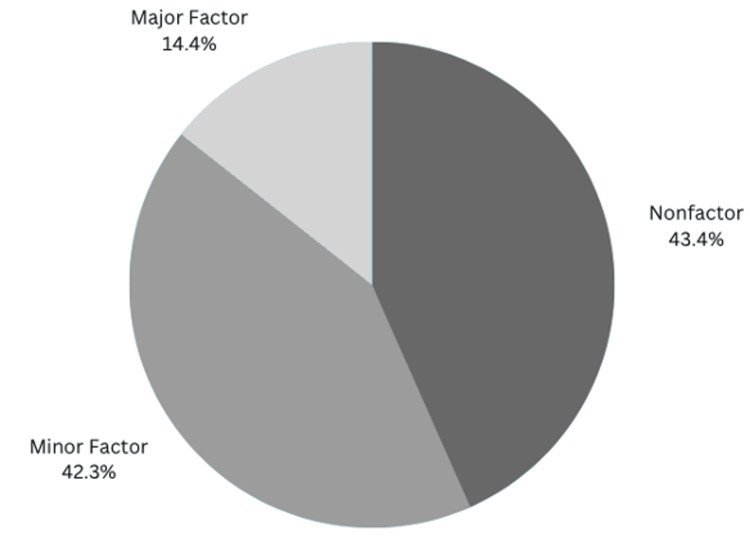
Percentage of medical students that cited medicolegal risk as "nonfactor", "minor", and "major" factor in influencing their decision to pursue Ob/Gyn Most medical students reported malpractice lawsuit risk as a “nonfactor” or “minor factor” in their chosen specialty, 43.4% and 42.3%, respectively. Meanwhile, only 14.4% reported it as a “major factor” influencing their chosen specialty. Fischer's exact test was used.

Regarding students’ perception of medico-legal risk as an influence on specialty choice, 43.4% considered it a non-factor, 42.3% a minor factor, and 14.4% a major factor (Figure 2). Notably, 79.4% of respondents had “slight” to “no” understanding of medical liability laws (Table 3).

**Figure 3:**
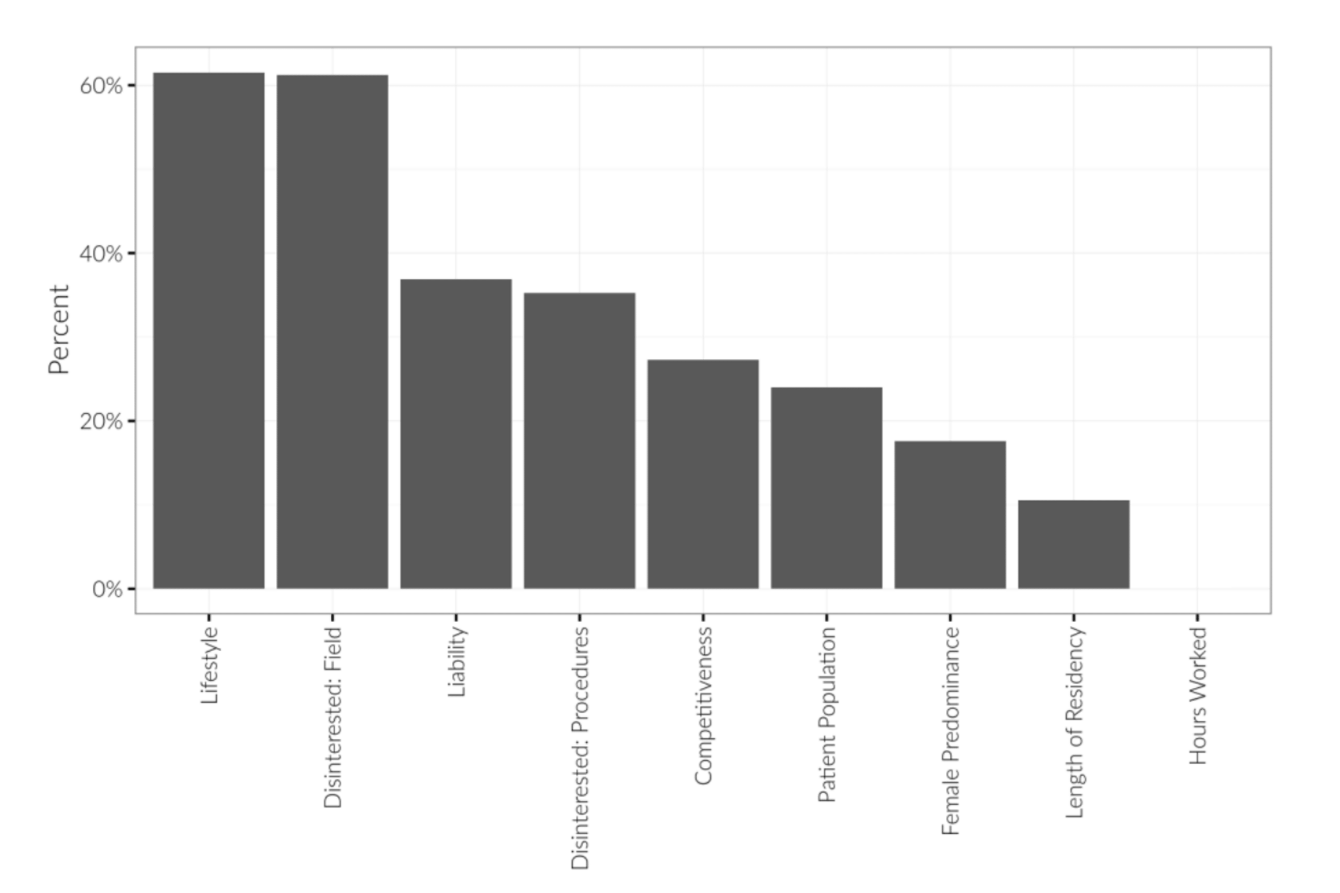
Reasons medical students reported for not pursuing Ob/Gyn This table shows the reported factors that deterred medical students from pursuing Ob/Gyn, where medical students had the option to list up to four reasons. The top four most common factors for students disinterested in Ob/Gyn were lifestyle, disinterest in the field, medical liability, and disinterest in procedures. Pearson's chi-squared test was used.

Regarding the factors against pursuing Ob/Gyn, medical liability ranked as the third highest reason (36.9%), with lifestyle (61.5%) and disinterest in the field (61.2%) as the top two. Other factors, including disinterest in procedures, competitiveness of the specialty, patient population, female predominance, length of residency, and hours worked, are shown in Figure 3. Additionally, gender-specific differences in reasons for not pursuing Ob/Gyn were evaluated. Females who did not choose Ob/Gyn as a specialty placed more emphasis on lifestyle and competitiveness, while male medical students were disinterested in the field due to patient population and female predominance. However, those who did choose to pursue Ob/Gyn were more likely to consider patient population, patient relationships, ability to perform procedures, and the prestige of the specialty.

## Discussion

Main findings

Our research findings reveal intriguing insights into the complex factors that influence medical students’ decision to pursue a career in Ob/Gyn, specifically how medical liability affects their decision. Contrary to expectations, medical liability risk does not appear to be a significant deterrent to selecting Ob/Gyn, although it is ranked as the third most common factor discouraging interest in the field. While the financial implications of malpractice risk are noteworthy considerations in any medical specialty, they were not the primary determinant nor significantly more influential among those choosing Ob/Gyn compared to those who did not.

Interestingly, Pyskoty et al.'s study found that 40% of medical students chose high-risk specialties despite awareness of litigation issues, citing enjoyment and procedural effectiveness as motivating factors for pursuing specialties like Ob/Gyn [11]. Additionally, other research suggests that malpractice has a negative predicted effect on specialty choice, while salary acts as a positive predictor. Furthermore, a study on malpractice risk by Anderson et al. highlights that physicians in high-risk specialties such as Ob/Gyn face nearly universal exposure to malpractice claims, yet this does not uniformly deter specialty selection.

Our findings align with the nuanced understanding presented in prior literature, indicating that while malpractice risk is acknowledged, it does not consistently outweigh the appeal of factors such as procedural opportunities, patient relationships, and the prestige associated with Ob/Gyn. This contrast underscores how individual motivations, such as the desire to make meaningful impacts in women’s health, may mitigate concerns about litigation. Our study’s strength lies in its focus on contemporary data and its ability to capture evolving trends in medical students’ specialty preferences, distinguishing it from earlier studies that may not reflect current educational and legal climates. However, limitations in geographic focus and demographic diversity of our sample may impact the generalizability of these findings, calling for further research to explore these dynamics in broader and more varied populations.

A study found that a significant proportion of male medical students believe their gender negatively impacts their educational experiences in Ob/Gyn. This perception is often reinforced by female patients who express a preference for female Ob/Gyn providers, potentially limiting male students' clinical exposure and opportunities [12]. Such biases not only deter male students from pursuing careers in Ob/Gyn but also perpetuate the gender imbalance within the specialty. These findings align with our study's observations regarding gender-related imbalances in Ob/Gyn. Male students in our research reported being deterred from the specialty due to patient population preferences and the perception of Ob/Gyn as a female-dominated field. Conversely, female students emphasized factors such as lifestyle and competitiveness in their decision-making process. Male medical students were also more likely to rate their knowledge of medicolegal risk as higher than their female colleagues. This study differs from previous studies in that prior studies included equal numbers of male and female medical student participants [1, 10, 11], whereas our study is more reflective of today’s landscape, where women have become the majority of medical school attendees since 2019 [13]. Given this, our study highlights a more realistic picture of the current disparities between male and female medical students as it relates to self-perceived knowledge of medicolegal risk. 

Addressing these gender disparities requires targeted interventions. Implementing mentorship programs that encourage male medical students to explore Ob/Gyn and educating patients about the competencies of all providers, regardless of gender, could help mitigate biases. Additionally, fostering an inclusive educational environment that challenges stereotypes and supports diverse career choices is essential for creating a balanced workforce in Ob/Gyn.

Additionally, a study analyzing factors influencing medical students' decisions to pursue Ob/Gyn in Germany found that while female graduates showed high interest, there was a declining proportion of male students choosing this specialty. This trend suggests that gender-specific factors significantly impact specialty choice, with male students possibly deterred by perceptions of Ob/Gyn as a female-dominated field [14]. Further research indicates that as certain specialties become increasingly female-predominant, they may experience a relative decline in earnings over time. This phenomenon, observed in the broader U.S. workforce, suggests that the feminization of a specialty can influence its economic valuation, potentially affecting both male and female physicians' decisions to enter these fields [15]. 

These findings underscore the need for targeted mentorship and educational interventions to address gender-specific deterrents and promote a more balanced and diverse workforce in specialties like Ob/Gyn. By understanding and addressing the underlying factors contributing to gender imbalances, the medical community can work toward creating a more equitable environment for all practitioners.

Strengths

Our findings align with the nuanced understanding presented in prior literature, indicating that while malpractice risk is acknowledged, it does not consistently outweigh the appeal of factors such as procedural opportunities, patient relationships, and the prestige associated with Ob/Gyn. This contrast underscores how individual motivations, such as the desire to make meaningful impacts in women’s health, may mitigate concerns about litigation. The strength of our study lies in its focus on contemporary data and its ability to capture evolving trends in medical students’ specialty preferences, distinguishing it from earlier studies that may not reflect current educational and legal climates. 

In addressing the pivotal question of whether malpractice risk significantly influences students’ decisions regarding Ob/Gyn, our findings challenge conventional assumptions. We found that medical liability concerns do not serve as a significant deterrent for those considering Ob/Gyn. Students appear to appreciate Ob/Gyn's unique attributes and its potential for meaningful impact, despite legal concerns. 

Limitations

This study is limited by its cross-sectional design, capturing a single point in time without accounting for changes over time. Additionally, fourth-year students were not surveyed after the residency match in March 2022, leaving it unknown whether those deterred from Ob/Gyn ultimately applied or matched. However, as our focus was on students' intended specialty choices, this does not significantly impact the validity of our findings. Future studies could compare intended specialties with actual match outcomes while considering medicolegal risks.

The survey measured students’ preferred specialty rather than their actual choice, leaving uncertainty about whether responses reflected aspirations or realistic decisions. The COVID-19 pandemic may have further influenced career choices, particularly for the graduating classes of 2023 and 2024, who faced disruptions such as online anatomy labs, potentially impacting their preparedness for surgical specialties. Additionally, virtual clinical rotations likely reduced Ob/Gyn exposure, limiting hands-on experience and understanding of the specialty.

The survey may not have fully captured all factors influencing interest in family medicine, though an open-ended response option helped mitigate this limitation. Timing may also have influenced responses, as the survey was conducted from January to February of 2022, a period when third-year students transition into new rotations, potentially increasing engagement, whereas fourth-year students, post-interviews and involved in away rotations, may have been less responsive.

Finally, response variability may reflect differences in medical school demographics, including rural versus urban settings, minority representation, and first-generation status, all of which could shape perceptions of medicolegal risks and specialty preferences.

Interpretation

This research offers valuable insights into the factors influencing medical students’ career decisions. Surprisingly, concerns regarding medical liability do not significantly deter this cohort of students from considering this specialty, underscoring their appreciation for its unique characteristics. Nevertheless, factors such as patient relationships and procedural opportunities resonate strongly with those drawn to Ob/Gyn, suggesting a deeper appeal beyond legal considerations. Our findings highlight the need for enhanced education on medical liability and the diverse array of factors shaping career trajectories within the medical field, as revealed through our survey of students at Western University across multiple graduating class years.

Despite limited exposure to medical liability education, respondents demonstrate varying levels of understanding, with the specialty’s appeal influenced by factors such as lifestyle, disinterest, and gender-specific perceptions, with previous studies showing similar responses from medical students [1,4,7]. Research indicates that concerns about malpractice risk and potential liability significantly influence specialty choice, particularly in high-risk fields such as obstetrics and gynecology, surgery, and emergency medicine. For example, medical students and residents may be deterred from pursuing these specialties due to fear of litigation, which is often more pronounced in fields with higher procedural volumes or direct patient risk [16,17].

Conversely, some students may still choose high-risk specialties due to factors such as lucrative salaries, large sign-on bonuses, and the opportunity to pay off student loans more quickly [18]. Additionally, lifestyle considerations, including predictable work hours or a more favorable work-life balance, play a major role, especially when balanced against the perceived stress of liability exposure [19]. The influence of mentors and clinical role models also shapes career decision-making, as positive mentorship can mitigate fears of liability through education and supportive networks.

Comparing these findings to our results suggests that liability concerns are one component of a complex decision-making process, where personal values, financial considerations, and mentorship experiences intersect. Addressing gaps in liability education could empower students to make more informed choices, potentially balancing risk perception with professional goals.

## Conclusions

In conclusion, this research study demonstrates that medical liability is not a significant determining factor in medical students' decision to pursue Ob/Gyn if they were already planning to do so. Gender-specific differences highlight the varied motivations for pursuing Ob/Gyn and the perceived knowledge of medical liability. The latter underscores gender disparities that exist within the medical education system and must be addressed. Gender-specific differences also emphasize the need for tailored educational and mentorship programs to reduce deterrents and foster diversity within the specialty.

By being aware of the factors that deter or attract medical students toward Ob/Gyn-and the gender disparities that exist in medical legal knowledge among medical students-medical schools can better support their students in their journey toward selecting a specialty. For those who chose Ob/Gyn, the results emphasize the value of patient relationships, procedural opportunities, and the specialty's prestige. Addressing these insights can inform the development of strategies to attract a more balanced and diverse workforce and enhance informed decision-making when considering a medical specialty. Further research is needed to explore the evolving landscape of specialty choices in a dynamic healthcare environment across broader populations and timeframes.
